# 

**Published:** 1890-03-22

**Authors:** 


SATURDAY, MARCH 22nd, 1890.

**Spending, Mending, or Ending** . . . 383
**Words of Consolation.**—Mourning . . . 384
**The Hospital Problem.** By Mr. Henry C. Burdett. III.—Hospital Audit and Bookkeeping . . . 385
**The Way they Died then** . . . 386
**The Doctor's Pulpit.**—A Sunday Lecturer . . . 387
**The Hospital for the Insane** . . . 388
**Everybody's Page** . . . 389
**Notes and News** . . . 390
**Annotations.**—Barmaids and their Friends—Inebriate Women and Girls—A Modern Mountebank . . . 392
**Turning Over New Leaves** . . . 396
**The Practitioner's Mirror and Retrospect:**
Medicine—Exophthalmic Goitre—Difficult Diagnosis . . . 393
Surgery—Hepatic Abscess . . . 394
Therapeutics — Naphthol in Dyspepsia—Acetanilide in Enteric Fever —Impurites of Salicylic Acid—Methyl-Acetandilide . . . 394
Practitioners' Bookshelf—Insomnia . . . 395
Dietetics—Acute Catarrh of the Stomach . . . 396
**The Hospital Workshop.** XXIII. — West of England Sanatorium . . . 397
**Passing Topics.**—Hampstead Provident Dispensary . . . 398
**Scraps and Gleanings** . . . 398

EXTRA SUPPLEMENT—THE NURSING MIRROR.

**En Passant.**—A Hint to Matrons—Burton Nursing Institution—Thousands of Pounds for the National Pension Fund—District Nursing at Gloucester—Cheering the Patients—Emigration—Dispensing . . . ci
**Nursing Lectures.** By Percy Lewis, M.D. V.—Chief Dangers of Chest Cases . . . cii
**Presentation** . . . civ
**Everybody's Opinion.**—The Duties of a Union Nurse—Idiot Cases—Who will help?—The Zenana Medical College . . . ciii
**Death in Our Ranks** . . . civ
**Notes and Queries** . . . civ
**Vacancies** . . . civ
**Amusements and Relaxation** . . . civ

